# H_2_-TPR Application for Sensitivity Analysis of In_2_O_3_-Based Nanostructure Layers

**DOI:** 10.3390/mi17080939

**Published:** 2026-08-06

**Authors:** Kirill S. Polunin, Mariya I. Ikim, Kairat S. Kurmangaleev, Varvara A. Demina, Olusegun J. Ilegbusi, Leonid I. Trakhtenberg

**Affiliations:** 1 N.N. Semenov Federal Research Center for Chemical Physics RAS, 4 Kosygin Street, 119991 Moscow, Russia; kpolunin62@gmail.com (K.S.P.); ikimmary1104@gmail.com (M.I.I.); f7033@mail.ru (K.S.K.); litrakh@gmail.com (L.I.T.); 2 Department of Mechanical and Aerospace Engineering, University of Central Florida, Orlando, FL 32816, USA; 3 Chemical Faculty, Lomonosov Moscow State University, 119991 Moscow, Russia

**Keywords:** indium oxide, adsorbed oxygen, hydrogen, temperature-programmed reduction, mechanical activation, sensitive layer, resistance, sensor response

## Abstract

The hydrogen temperature-programmed reduction (H_2_-TPR) method was used to analyze the sensing properties of nanostructured indium oxide for hydrogen detection. Commercial and mechanically activated In_2_O_3_ samples were selected for investigation. Mechanical activation leads to the generation of surface defects and an increase in specific surface area, which enhances sensitivity to H_2_ and lowers the sensor operating temperature. An approach is proposed that allows a qualitative and quantitative relationship to be established between the H_2_-TPR profiles of the oxides and the sensor response. This relationship is based on a model of electron transfer across a potential barrier at grain boundaries, formed with the participation of adsorbed oxygen. The temperature dependence of the sensor response is found to be determined by the concentration of negatively charged oxygen on the surface of the nanoparticles, which, in turn, depends on temperature. Using the sensor sensitive layer based on indium oxide as an example, a correlation is established between the parameters of the TPR profiles and the sensor response.

## 1. Introduction

Gas sensors based on metal-oxide semiconductors (MOS) are among the most widely used devices for detecting toxic, flammable, and explosive gases in the environment. Their advantages include compact size, simple design, and the ability to operate over a wide range of gas concentrations [1]. Most of the modern gas sensor devices are based on metal oxide semiconductors [2,3,4]. Indium oxide plays an important role among the materials used for sensing layers. Sensors based on In_2_O_3_ demonstrate high sensitivity and fast response to a wide range of reducing and oxidizing gases, including hydrogen, carbon monoxide, hydrogen sulfide, and ozone [5,6,7,8,9]. These properties make In_2_O_3_ a promising material for conductometric gas sensors.

A distinctive feature of In_2_O_3_ as a semiconductor is the shallow depth of donor level, which leads to a high concentration of conduction electrons. At the same time, the functional characteristics of sensors based on this oxide are governed by surface processes involving oxygen adsorption at vacancies, the formation of charged oxygen species, and subsequent reactions with target gases. Oxygen anion radicals and oxygen vacancies play an important role in surface chemistry and affect the charge carrier concentration in semiconductors [10,11].

A key factor affecting the electrical properties of polycrystalline metal oxides—and consequently their sensing performance—is charge transport across grain boundaries, where adsorption of oxygen and formation of O^−^ species lead to potential barrier formation [12]. The height of these barriers depends on the concentration of adsorbed oxygen species, making conductivity highly sensitive to the gas environment.

Previously, the hydrogen temperature-programmed reduction (H_2_-TPR) method was applied to investigate processes occurring on the surfaces of various samples, including those in the H_2_/In_2_O_3_ system [13,14,15]. However, this approach has been used primarily for the characterization of catalysts for chemical reactions. Its application for the quantitative description or prediction of gas-sensing characteristics has not been previously discussed.

It is well-known that the state of the sensing layer determines the kinetics of gas interactions with surface reaction centers and consequently affects the sensing performance of the material. Several studies have investigated the surface properties of metal oxides using various methods and described their influence on sensor response (e.g., [16,17,18,19,20,21]).

H_2_-TPR, in contrast to widely used techniques that characterize the surface under steady-state conditions (e.g., XPS, DRIFTS, and EPR), enables investigations under conditions that are close to the actual operating regime of a gas sensor. In addition, this technique provides the temperature dependence of the rate of the reduction process. These advantages make H_2_-TPR a suitable method to assess sensor characteristics such as the maximum sensor response and the optimal operating temperature for gas detection.

An important factor determining both the reduction kinetics and the sensing properties of oxide semiconductors is the initial surface state formed during sample pretreatment. Different processing conditions of indium oxide result in a variety of initial surface states, which are reflected in the structure of TPR profiles. Analysis of such profiles allows the separation of contributions from different surface reaction centers, including oxygen species and possible impurities of various types, as well as distinguishing surface and bulk processes. Such separation is necessary for the correct interpretation of the relationship between TPR data and sensor performance.

This study proposes a procedure that links TPR profiles with the temperature dependence of sensor response. This approach enables the establishment of the transition from surface reaction kinetics to the formation of grain-boundary potential barriers within a specific conductivity model [12], and consequently to changes in sensing properties. The procedure can be used as a predictive methodology for the evaluation of conductometric sensor performance based on TPR data of the surface.

## 2. Materials and Methods

Commercial and mechanically activated indium oxide were used as sensing materials to assess the applicability of the TPR method for evaluation hydrogen response in metal-oxide sensors. In_2_O_3_ powder (LANHIT, Moscow, Russia, 99.99% purity) was utilized, and mechanical milling was performed using a high-energy Aronov mill (Moscow, Russia) [22]. Samples were subjected to 30 min of milling. The materials were designated as In_2_O_3_(0 min) and In_2_O_3_(30 min).

Phase composition and structural properties were studied by X-ray diffraction (XRD) using a Rigaku SmartLab diffractometer (Rigaku, Tokyo, Japan) with monochromatic Cu(Kα) radiation (λ = 1.5406 Å). Scanning was performed in the 2θ range from 15° to 80° at a rate of 5°/min. Particle morphology was analyzed by scanning electron microscopy (SEM) using a Prisma E microscope (Thermo Fisher Scientific, Waltham, MA, USA).

Specific surface area was determined by the Brunauer–Emmett–Teller (BET) method at a relative pressure *P*/*P*_0_ = 0.30 using an AMI-400TPx system (Altamira Instruments, Pittsburg, PA, USA). Temperature-programmed reduction (TPR) with hydrogen was conducted on the same setup. Prior to measurements, samples were treated in an argon flow at 200, 300, 400, 500, and 700 °C for 10 min, then cooled to 50 °C in inert gas. Reduction was carried out in 10 vol% H_2_ in Ar at a flow rate of 30 mL/min up to 500 °C with a heating rate of 10 °C/min. Experimental values of the TCD detector signal during reduction were recorded every 0.5 s.

For sensor fabrication, powders were mixed with terpineol to form a paste, which was then deposited onto commercial alumina substrates equipped with platinum heaters and electrodes for resistance measurements [23]. The substrates were subsequently heated to 550 °C to stabilize film resistance. Three sensors were fabricated based on commercial and mechanically activated powders. Electrical conductivity and gas-sensing properties were measured using a custom setup in the temperature range 300–550 °C [23]. Sensor response was defined as the ratio $R_{air}/R_{gas}$, where $R_{air}$ and $R_{gas}$ are respectively the resistance of the sensing layer in air and in 900 ppm H_2_. Resistance was measured using a Keithley 34465A 6^1/2^ (Keysight, Santa Rosa, CA, USA) multimeter.

## 3. Results and Discussion

### 3.1. Structural and Morphological Studies

**Figure 1:**
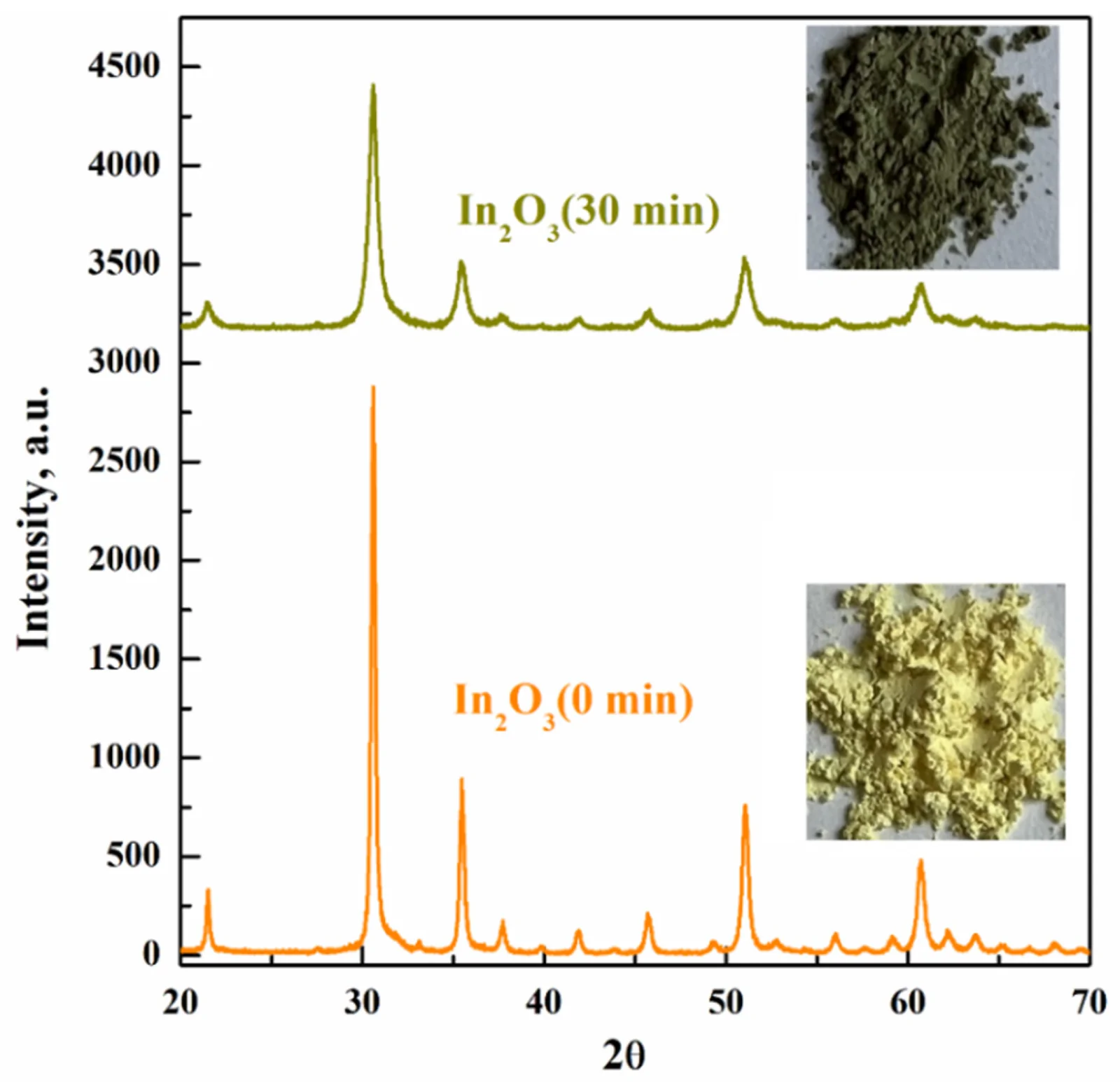
XRD spectra of commercial In_2_O_3_ powder and that subjected to mechanical activation for 30 min. The inserts show the appearance of the samples.

The phase and structural characteristics of the model samples were investigated by X-ray diffraction (XRD) analysis. The XRD patterns of the samples exhibit characteristic diffraction peaks corresponding to the cubic structure of In_2_O_3_ (JCPDS card No. 71-2194) (Figure 1). The absence of additional peaks in the diffraction patterns indicates the absence of impurity phases in the samples.

Mechanical activation leads to a decrease in the intensity of the diffraction peaks and their broadening. A similar trend has also been reported for other mechanically activated systems [24,25,26]. These changes are most likely associated with the accumulation of microstrains and defects generated during the milling process, as well as with a reduction in nanoparticle size.

Mechanical activation for 30 min also resulted in a visible color change of the powder from yellow to gray (see Figure 1 inset), which may be attributed to the formation of oxygen vacancies and structural defects [27,28,29]. The lattice parameter for In_2_O_3_(30 min) (10.118 Å) was higher than that of commercial In_2_O_3_ (10.115 Å), indicating increased internal strain and partial structural disorder.

In addition, the milling results in a reduction in particle size. Specifically, the Debye–Scherrer method indicates a decrease from 36 to 16.3 nm, while the Williamson–Hall method gives a reduction from 51.9 to 19 nm, respectively. BET analysis revealed an increase in the specific surface area (*S_BET_*) from 10 to 24 m^2^/g. Furthermore, this parameter was also determined for the In_2_O_3_(0 min) and In_2_O_3_(30 min) samples after argon treatment at 300 and 500 °C.

**Figure 2:**
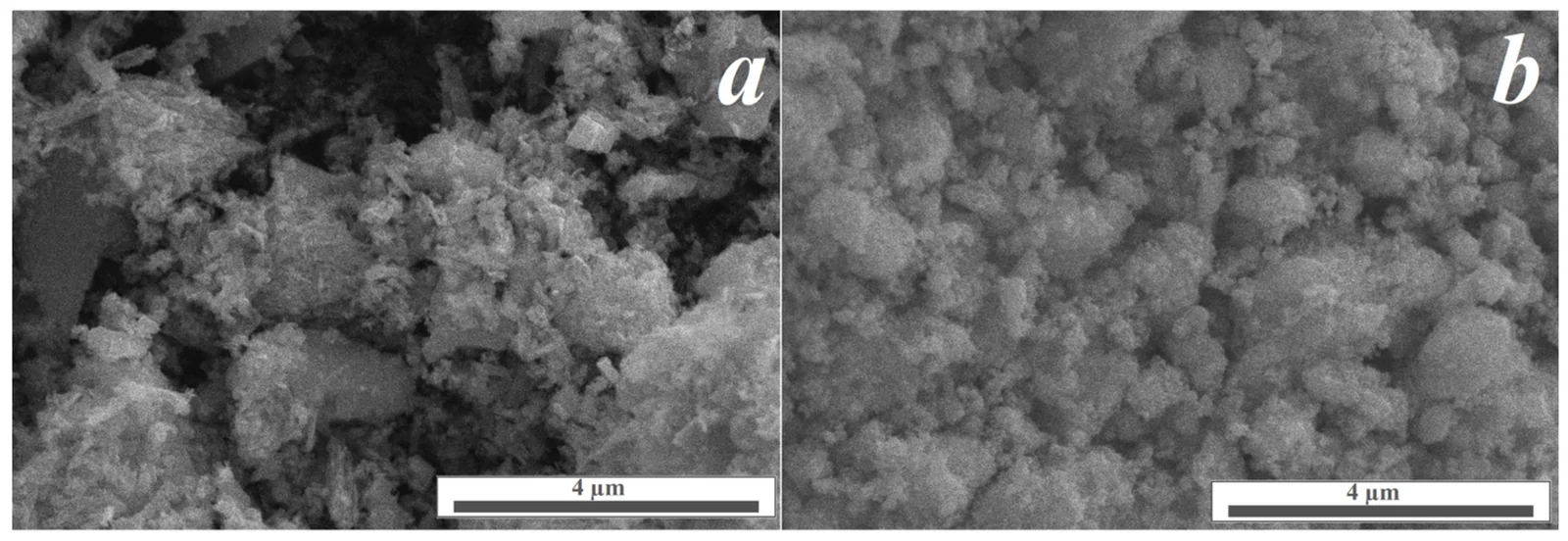
SEM images of commercial indium oxide powder (**a**) and that subjected to mechanical activation for 30 min (**b**).

The scanning electron microscopy results demonstrate that milling in the Aronov mill also leads to changes in the morphology of indium oxide particles (Figure 2).

The commercial In_2_O_3_ nanopowder is characterized by heterogeneity in shape and size of particles (Figure 2a). Both elongated particles with well-defined facets—lengths of hundreds of nanometers and an aspect ratio of about 4—and cubic-shaped particles with sizes less than 1 μm are observed (Figure 2a). In addition, large agglomerates of irregular shape with sizes up to 10 μm are detected. They are represented both by structures with rough surfaces and denser particles with smooth surfaces. Mechanical treatment leads to homogenization of the powder (Figure 2b). A reduction in the number of large aggregates is observed, while the fraction of individual particles increases, with sizes reaching 20 nm.

### 3.2. H_2_-TPR Profiles

**Figure 3:**
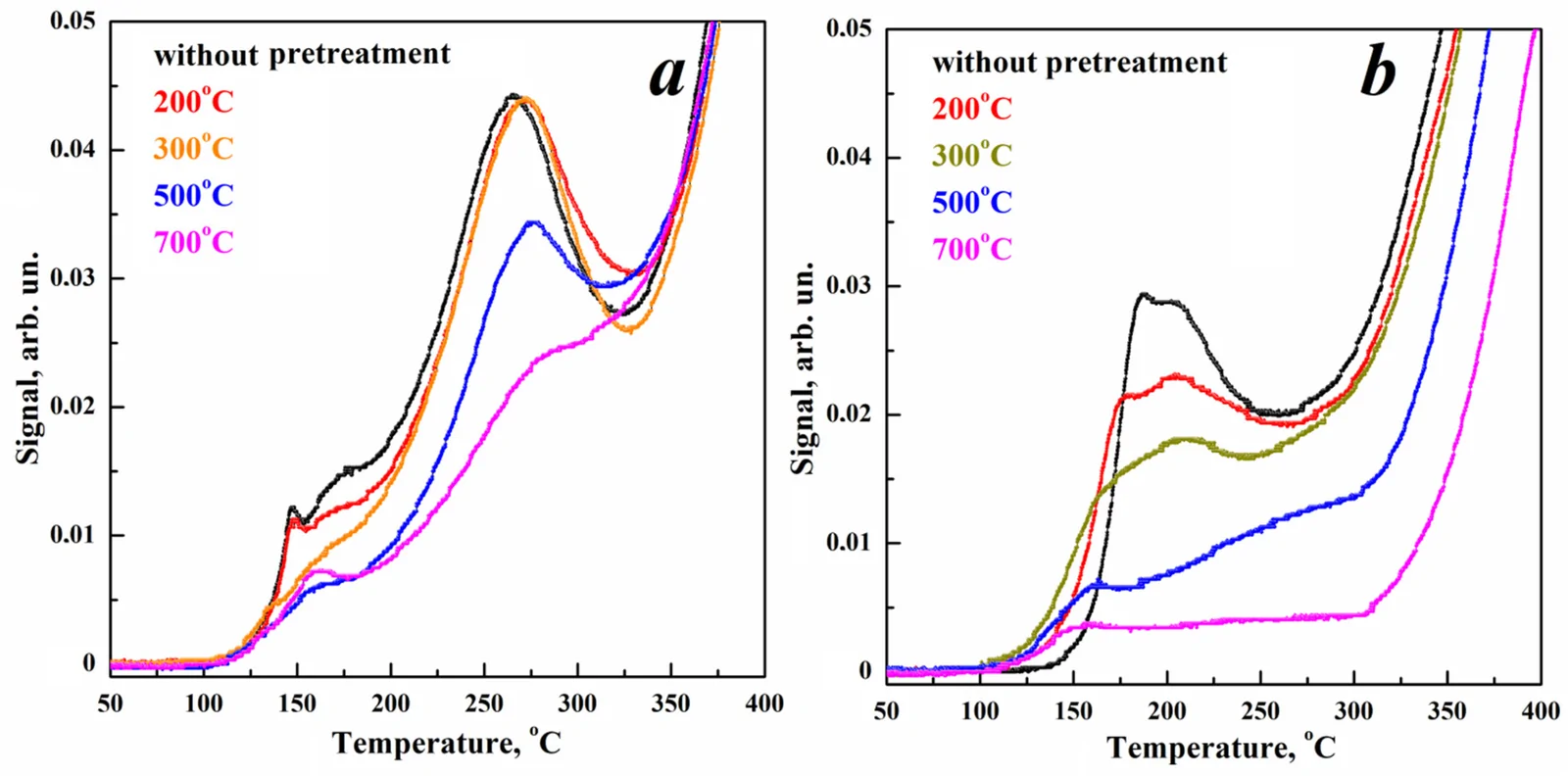
H_2_-TPR curves of In_2_O_3_(0 min) (**a**) and In_2_O_3_(30 min) (**b**) at different argon pretreatment temperatures.

A series of TPR experiments was conducted for both mechanically activated and untreated indium oxide samples. The varying parameter in this case is the temperature of pretreatment of the samples in the inert gas. The results are presented in Figure 3.

Figure 3a shows TPR profiles of pristine indium oxide at different pretreatment temperatures in inert gas. Two peaks are observed for the sample without pretreatment (black curve), corresponding to maxima in the rate of hydrogen reaction with the In_2_O_3_ surface. The appearance of a low-intensity peak at a temperature of about 150 °C is associated with the presence of impurities. This observation can be confirmed by analyzing the TPR profiles of indium oxide exposed to an oxygen-containing atmosphere.

**Figure 4:**
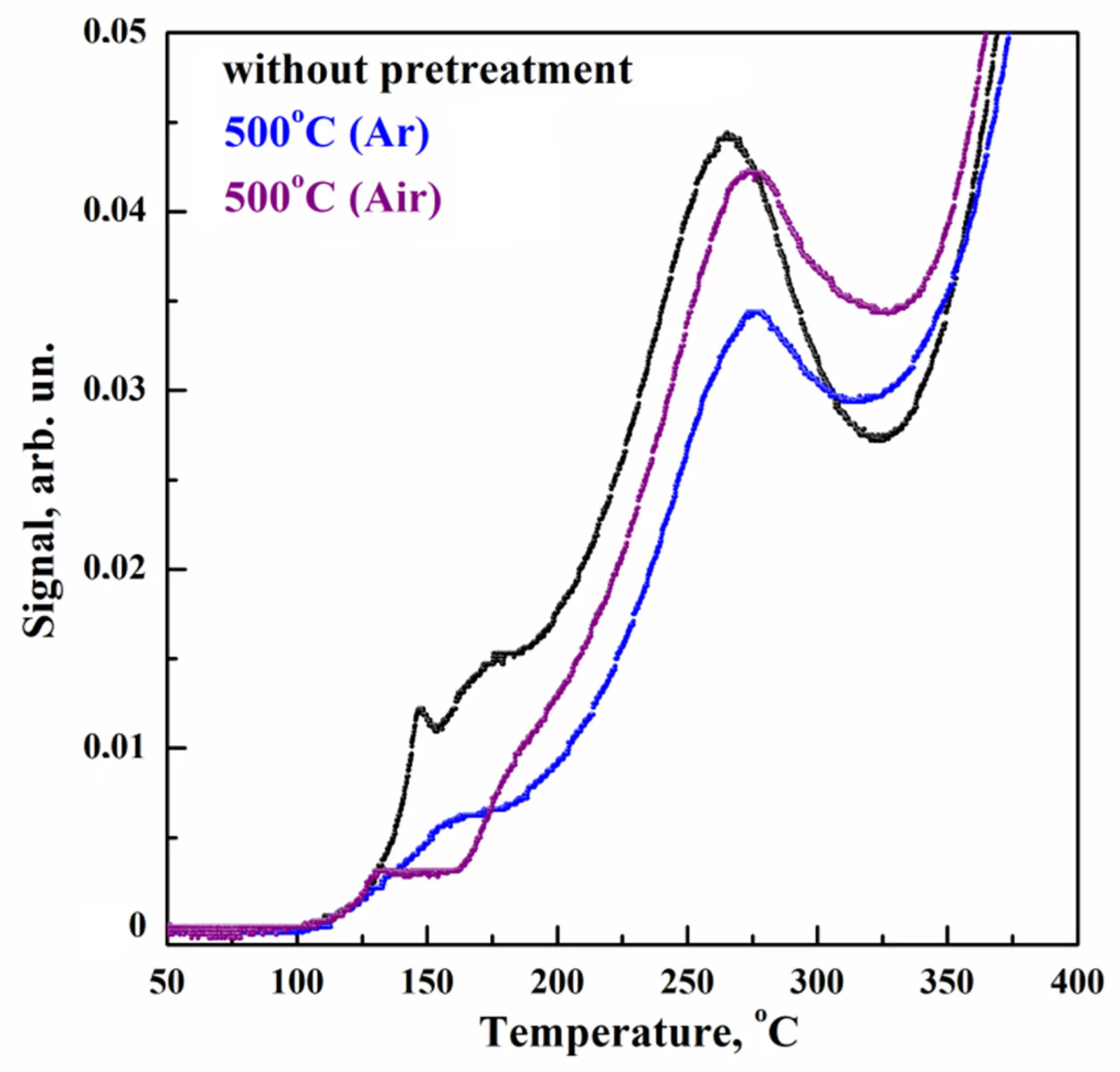
Temperature-programmed reduction profiles of indium oxide samples without mechanical activation subjected to thermal treatment at 500 °C in flows of different gases.

Figure 4 indicates that a compound (or a group of compounds) responsible for the maximum hydrogen interaction rate at a temperature of approximately 150 °C is removed during argon exposure via desorption. When treated under the same conditions in an oxygen atmosphere, a more pronounced decrease in the intensity of this peak is observed. This result indicates that in addition to desorption, impurities are also removed via chemical reactions. The impurities are most likely carbon-containing residuals after synthesis, which can interact with hydrogen at relatively low temperatures (100–200 °C). The narrow shape of the peak indicates a limited number of these groups on the surface. A stronger suppression of the signal in oxygen confirms that this maximum is not related to the interaction of hydrogen with surface oxygen species and does not contribute to the sensor effect.

It should be noted that treatment in air does not lead to a decrease in the concentration of surface oxygen species, which are removed via desorption. At the same time, oxygen vacancies formed are effectively refilled in the presence of O_2_.

The sample may also contain compounds that do not interact with oxygen but react with hydrogen. These include, for example, hydroxyl groups formed during storage of In_2_O_3_ powder in a humid atmosphere. Such groups are removed mainly by thermal desorption and can be effectively eliminated both in inert and oxidizing environments.

This is consistent with the results presented in Figure 4. The maximum intensity of the main peak (~270 °C) is observed for the untreated sample. After thermal treatment in air, its intensity decreases, which is associated with removal of impurities while preserving oxygen centers. The minimum signal is observed after argon treatment, which simultaneously leads to the removal of impurities and a decrease in surface oxygen concentration.

After elimination of the impurity contribution, the kinetics of hydrogen interaction with In_2_O_3_ are mainly determined by the interaction with surface oxygen centers. The following sequence is observed in the absence of pretreatment: impurity peak at ~150 °C, main surface reduction peak at ~270 °C, and transition to the bulk reduction region.

The surface state changes from argon treatment are due to the removal of impurities and parts of weakly bound oxygen groups. This leads to the disappearance of the low-intensity peak and a decrease in the intensity of the main peak corresponding to surface reduction. The latter effect is enhanced with increasing pretreatment temperature.

Mechanical activation significantly changes the surface of In_2_O_3_ and the kinetics of hydrogen reduction. TPR profiles for mechanically activated samples are shown in Figure 3b. The impurity peak observed for pristine systems is absent in the curves for mechanically activated samples. This indicates removal of surface carbon-containing impurities that previously enabled interaction with hydrogen at about 150 °C.

At the same time, the concentration of structural defects, including oxygen vacancies, increases, which changes the kinetics of interaction with hydrogen. The most pronounced evidence of this effect is the splitting of the main surface reduction peak. Instead of a single maximum at 270 °C, two overlapping peaks at lower temperatures are observed. Such behavior indicates the presence of at least two types of surface or near-surface active centers with close but different activation energies for reaction with hydrogen.

A region of nearly constant rate of gas–surface interaction appears after pretreatment at 700 °C. This feature is observed in the temperature range of 150–300 °C for mechanically activated samples. A similar region is detected over a narrower range of 150–170 °C for non-mechanically activated nanoparticles. The emergence of a constant-rate region is associated with a transition of the system into a quasi-steady state, in which the observed interaction rate is determined by the balance between consumption of surface oxygen and its replenishment (e.g., due to diffusion from the bulk). This state is characterized by a weak temperature dependence of the observed signal. The appearance of such a region in the TPR profile indicates that argon pretreatment at 700 °C almost completely removes reactive oxygen species from the surface.

For the investigation of the sensor process, it is recommended to subject the material to pretreatment at a lower temperature. The optimal conditions in this case can be considered as those under which the impurity contribution is minimized while enough oxygen centers remain on the surface. As Figure 3 indicates, a temperature of 300 °C is sufficient to remove most impurities. According to BET measurements, argon pretreatment of the pristine powder at 300 °C did not significantly change the *S_BET_* value, as was also observed for the mechanically activated sample. The crystallite size, calculated from the (222) diffraction peak, also remained nearly constant in both cases. On the other hand, argon treatment at 500 °C led to sintering of In_2_O_3_(30 min). That resulted in a pronounced decrease in the specific surface area from 24 to 7 m^2^/g and an increase in the crystallite size from 16.4 to 25 nm. Such an effect was not observed for the pristine sample. Therefore, comparison of pristine and mechanically activated samples becomes unreliable. Consequently, TPR profiles obtained for indium oxide pretreated in argon at 300 °C were used to verify the model introduced in the following sections.

### 3.3. Temperature Dependences of Sensor Response

**Figure 5:**
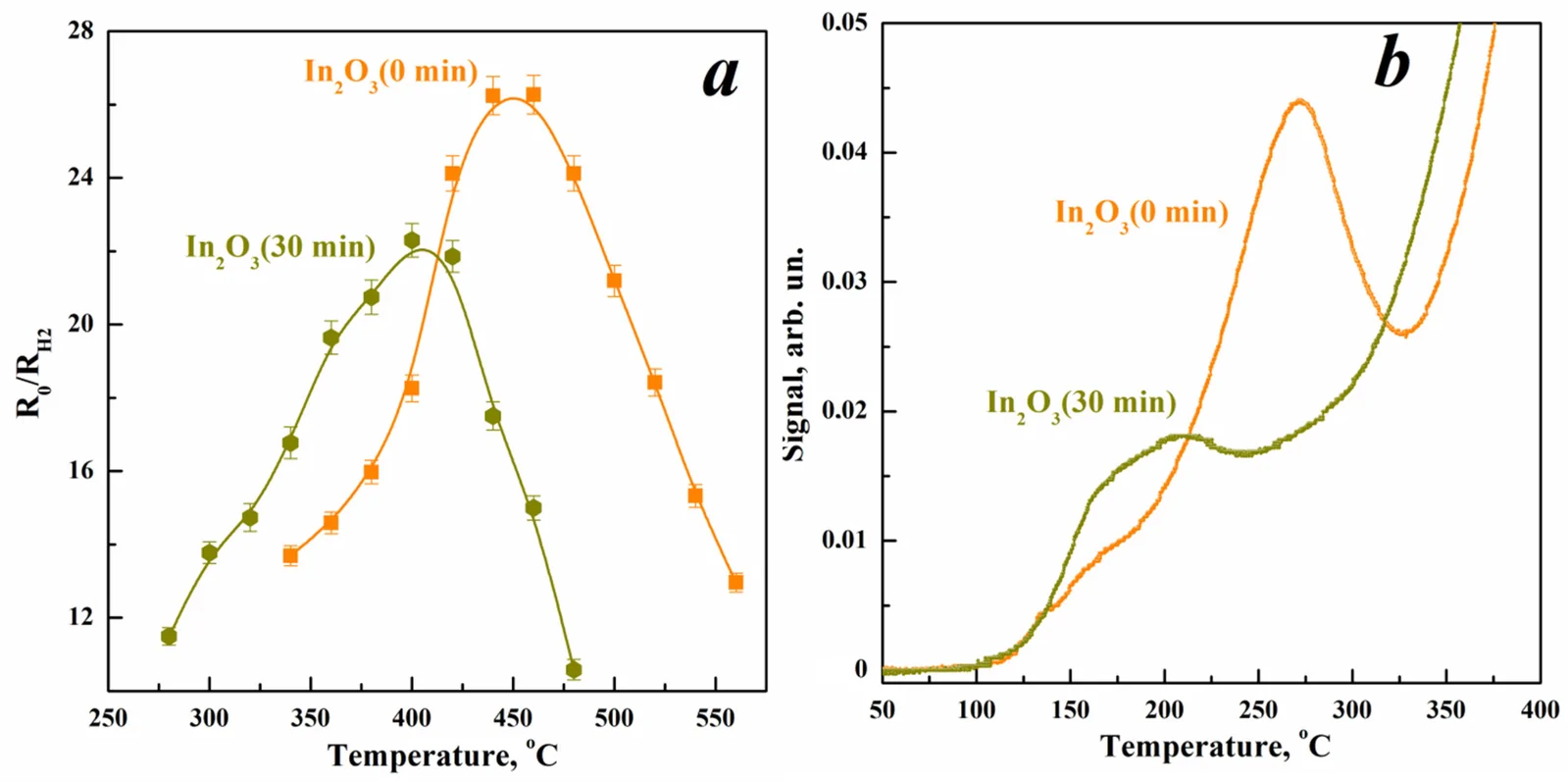
Temperature dependence of the sensor response to 900 ppm H_2_ of In_2_O_3_(0min) and In_2_O_3_(30min) (**a**), and H_2_-TPR curves at pretreatment temperature 300 °C of In_2_O_3_(0min) and In_2_O_3_(30min) (**b**). The error bars in (**a**) represent the standard deviation of the sensor response measured using three independently fabricated sensors.

To characterize the sensing properties of In_2_O_3_, a series of measurements of its response to hydrogen was carried out. Since the sensing effect is based on surface reduction reactions, the obtained data were compared with TPR profiles (Figure 5).

The results from measurement of the sensor response of indium oxide to hydrogen at different temperatures are presented in Figure 5a. These results confirm that mechanical activation decreases the maximum achievable sensor response, while the characteristic temperature of the maximum is shifted to lower values.

At the same time, mechanical treatment leads to a faster surface reduction rate due to the decrease in particle size and the increase in specific surface area. Specifically, the In_2_O_3_(0 min) samples are characterized by a relatively long response time of 2.2 s and a slow recovery time of 68.0 s, while the In_2_O_3_(30 min) samples demonstrate significantly improved dynamic characteristics, with a response time of 0.9 s and a recovery time of 8.6 s. In addition, the reproducibility and stability of the In_2_O_3_(30 min) sensing layer were evaluated at the operating temperature under exposure to 900 ppm H_2_. The mechanically activated sample maintained a stable response magnitude and consistent response and recovery times over seven consecutive hydrogen exposure/recovery cycles. Furthermore, the sensor response varied by no more than 5% over a period of three months. These results indicate that the In_2_O_3_(30 min) samples not only provide a faster response to hydrogen but also exhibit excellent reproducibility and long-term stability.

A similar change can also be observed in the TPR profile (Figure 5b). It was previously noted that the decrease in the intensity of surface reduction is due to an increase in defect concentration. Thus, the values of the sensor response and the TPR signal are related. Changes in the surface state led to the same effects in both experiments. Therefore, TPR profile parameters can be used to estimate changes in the sensing properties of the material.

### 3.4. Mechanism of Sensor Interaction

There are defects in the structure of indium oxide among which oxygen vacancies on the surface of nanoparticles play a key role in the sensing properties. When these vacancies are filled with molecular oxygen from air, adsorbed oxygen atoms capture electrons and form surface oxygen anion radicals. Such particles can exist in two forms: a weakly bound adsorbed form ($O_{ads}^{-}$) and a form that is incorporated into the crystal structure lattice ($O_{lat}^{-}$) [30]. At the beginning of the experiments, the surfaces of nanoparticles of indium oxide are saturated with oxygen anion radicals due to long-term exposure to air. High-temperature exposure to inert gas leads to the removal of adsorbed oxygen, as well as the release of adsorption centers, which promotes the transition $O_{lat}^{-}\to O_{ads}^{-}$ and formation of vacancies in the crystal lattice.

Hydrogen reacts with oxygen anion radicals on the surfaces of indium oxide nanoparticles [30]. Electrons transferred from $O_{ads}^{-}$ and $O_{lat}^{-}$ to the conduction band generate the sensor signal. Adsorption centers that are cleared during the process can be filled again with lattice oxygen atoms, thereby replenishing reactive oxygen centers.

### 3.5. Relationship Between TPR Signal and Sensor Response

The resistance (R) of polycrystalline oxides in this study is described within a thermally activated model [12], in which R is determined by the height of potential barriers at grain boundaries ($E_{b}$):

(1) $$E_{b}\;=\;e^{2}{\left(N_{O^{-}}\right)}^{2}/2\varepsilon \varepsilon_{0}N_{d}.$$

Here ε is the relative dielectric permittivity of the material; $\varepsilon_{0}$ is the electric constant; e is the elementary charge; $N_{O^{-}}$ is the concentration of surface oxygen anion radicals; and $N_{d}$ is the bulk concentration of charge donors.

The sensor response (*θ*) is defined as the ratio $\theta =R_{air}/R_{gas}$, where $R_{air}$ and $R_{gas}$ are respectively the resistance of the sensing layer in air and the target gas atmosphere. The thermally activated nature of conductivity relates resistance to barrier height via the Arrhenius-type equation, where $R\;\sim \;exp\left(E_{b}/k_{B}T\right)$. Thus, the sensor response can be expressed as:

(2) $$\theta =\frac{R_{air}}{R_{gas}}=\;exp\left(\frac{E_{air}\;-\;E_{gas}}{k_{B}T}\right).$$

Here $E_{air}$ and $E_{gas}$ denote the barrier heights at grain boundaries in air and the target gas atmosphere respectively; $k_{B}$ is the Boltzmann constant; and T is the absolute temperature.

The TPR profile reflects changes in the concentration of surface reaction centers ($\Delta N_{ads}$). Combining relations (1) and (2), and assuming that the change in concentration is small compared to the initial amount of ions ($\Delta N_{ads}\ll N_{O^{-}}^{air}$), it can be readily shown that:

(3) $$\theta (T)\;=\;exp\left(\frac{e^{2}}{2\varepsilon \varepsilon_{0}N_{d}k_{B}}\frac{N_{O^{-}}^{air}\left(T\right)\Delta N_{ads}\left(T\right)}{T}\right).$$

For further analysis, it will be assumed that $N_{O^{-}}^{air}\left(T\right)$ changes insignificantly with increasing temperature. This assumption follows from the near-saturated surface coverage with oxygen species under the conditions considered. In this regime, the concentration of available oxygen anion radicals remains approximately constant, and the temperature-induced changes in sensor response are mainly associated with the kinetics of the surface reaction.

It should be noted that direct comparison between dynamic TPR profiles and the sensor response measured under steady-state conditions requires careful consideration. Such an approach is valid when the reduction process occurs under quasi-steady-state conditions, for which the following requirements must be satisfied:

1. The characteristic time of temperature change is significantly longer than the characteristic time of the surface reactions, implying a relatively low heating rate;
2. Hydrogen consumption during the TPR experiment does not lead to significant local changes in its partial pressure;
3. The oxide microstructure remains stable within the analyzed temperature range.

Within these constraints, the process studied in the TPR experiment may be considered a sequence of quasi-steady states, enabling correlation of the obtained profiles with the temperature dependence of the sensor response.

To isolate the component corresponding to the sensing process (surface interactions excluding impurity contributions), it is convenient to perform decomposition of the TPR profile into Gaussian components. The H_2_-TPR signal is proportional to the rate of hydrogen interaction with the metal oxide surface. Its intensity corresponds to the change in the number of hydrogen molecules in the gas mixture over time. According to the gas-sensing mechanism described in Section 3.4, water molecules are formed in the reaction of gaseous hydrogen with surface oxygen anion radicals in a 1:1 ratio. Thus, the H_2_-TPR signal also reflects the change in the number of surface oxygen anion radicals consumed during the reaction.

Therefore, the change in concentration on the In_2_O_3_ surface can be determined as the integral of the TPR signal. Thus, $\Delta N_{ads}\left(T\right)$ can be expressed as:

(4) $$\Delta N_{ads}\left(T\right)\;=\;A\int_{T_{0}}^{T^{\prime }}exp\left(-\frac{{\left(T\;-\;T_{m}\right)}^{2}}{2\sigma^{2}}\right)dT.$$

Here A is the height of the Gaussian function, i.e., the TPR signal magnitude at the corresponding maximum; $T_{m}$ is the mean value, i.e., the temperature corresponding to the maximum reduction rate; and σ is the standard deviation characterizing the width of the distribution of surface reaction groups. $\Delta N_{ads}$ is a function of the set of parameters, so $\Delta N_{ads}$ = $\Delta N_{ads}(A,\sigma ,T_{m},T)$.

**Figure 6:**
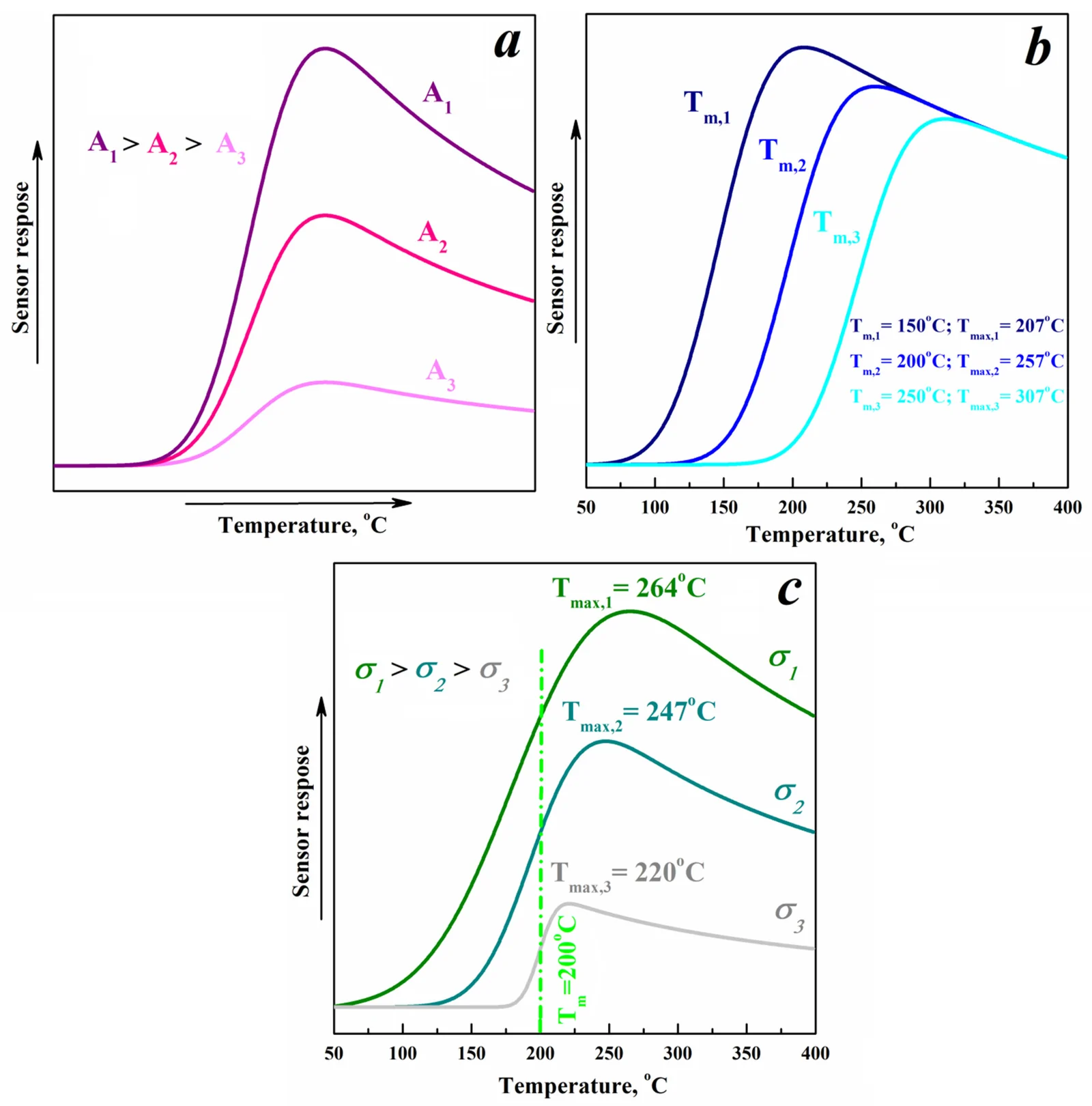
Influence of height (**a**), mean value (**b**) and standard deviation (**c**) of the Gaussian on the temperature dependence of the sensor response.

The following values were used to estimate the sensor response: ε = 4.88 [31]; $\varepsilon_{0}$ = 8.85 × 10^−12^ F/m; $N_{O^{-}}$ = 10^17^ m^−2^ [32]; $N_{d}$ = 10^25^ m^−3^ [33]. The influence of parameters A, $T_{m}$ and σ on the temperature dependence of the sensor response is shown in Figure 6.

Figure 6 shows that variation of parameter A has a significant effect on the absolute value of the sensor response but does not change the position of the maximum. On the other hand, parameters $T_{m}$ and σ affect both characteristics. The magnitude of the temperature shift of the maximum is determined only by the value of the standard deviation σ.

The proposed approach makes it possible to assess gas sensor characteristics without performing time-consuming isothermal sensitivity measurements. Specifically, a shift in the TPR peak toward lower temperatures ($T_{m}$ decrease), combined with a higher surface reduction rate (A increase), indicates a lower optimal operating temperature and a higher maximum sensor response. Furthermore, the width of the TPR peak, characterized by σ, can be used to evaluate the sensor operating temperature range. Therefore, the parameters extracted from TPR curves can be used to compare the sensing properties of semiconductor oxides.

To ensure that the system remains in a quasi-steady state, the sample must be heated sufficiently slowly that the *τ*—time required to pass through the characteristic temperature interval ΔT—exceeds the equilibration time in the investigated system. The temperature change over a period of time at a given heating rate β can be expressed as $\Delta T=\beta \tau \;=\;k_{B}T^{2}/E_{a}$. Such an estimate yields the values of ΔT ≈ 19.3–39 K, given that the activation energy ($E_{a}$) of the gas–surface reaction is approximately 1 eV [30], while T corresponds to the optimal operating temperature of the sensor (which is 200–400 °C).

The characteristic time required for the system to reach a quasi-steady state is equal to the response time of the sensor, which has a maximum value of 2.2 s, as mentioned before (see Section 3.3), and is significantly higher than the relaxation time. Using this value as *τ* together with ΔT = 19.3 K yields β of approximately 500°/min. At heating rates below this value, the sample may be considered heated sufficiently slowly to maintain a quasi-steady-state regime.

The heating rate used in the experiments, 10°/min, satisfies this criterion. Thus, the characteristic equilibration time of the surface reactions remains shorter than the characteristic heating time throughout the investigated temperature range. This consideration supports the quasi-steady-state approximation and justifies the use of TPR profiles to establish semi-quantitative correlations with the temperature dependence of steady-state sensor response.

### 3.6. Model Validation

**Figure 7:**
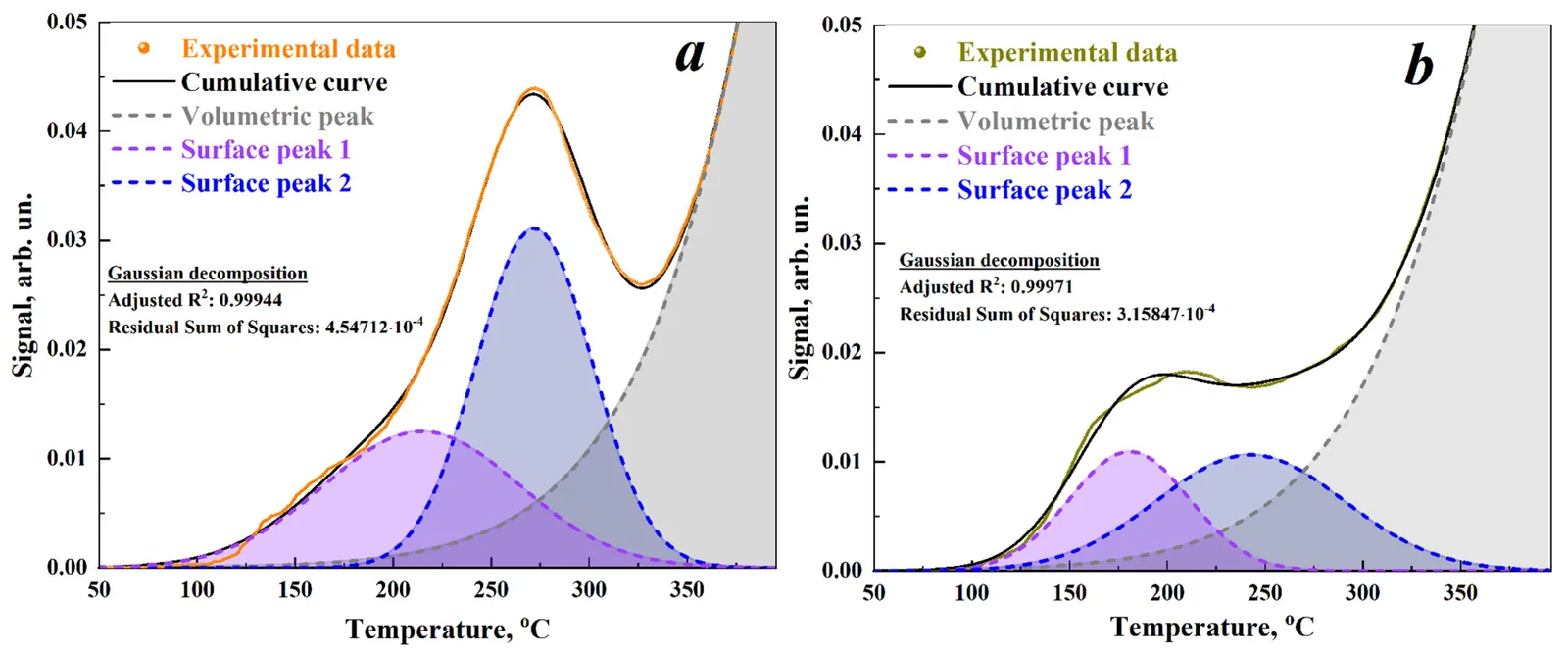
Decomposition into Gaussian components of TPR curves obtained for samples In_2_O_3_(0 min) (**a**) and In_2_O_3_(30 min) (**b**) pretreated in argon at 300 °C.

The TPR profiles of In_2_O_3_(0 min) and In_2_O_3_(30 min) samples treated in argon at 300 °C were analyzed to verify the model developed. These profiles were decomposed into Gaussian components (Figure 7), for which height, standard deviation, and mean value were determined (Table 1) using the Levenberg–Marquardt algorithm [34]. From Table 1, changes in Gaussian parameters can be observed and compared with the results of sensor response measurements (Figure 5a).

The surface interaction responsible for the sensing effect is described sufficiently accurately by two Gaussian peaks corresponding to the interaction of hydrogen with oxygen anion radicals in different states on the surface. As discussed in Section 3.4, two types of surface oxygen anion radicals are present on the In_2_O_3_ surface. The concentration of the weakly bound surface oxygen species ($O_{ads}^{-}$) is lower than that of $O_{lat}^{-}$. Accordingly, the reduction of adsorbed oxygen anion radicals produces the less intense low-temperature reduction peak (Peak 1 in Table 1).

It is remarkable that the parameters of the related Gaussian component change only slightly after mechanical activation. This result implies that the sensing effect is mainly influenced by changes in the reduction conditions corresponding to the higher-temperature maximum in the TPR curve.

The intensity of the main peak observed for In_2_O_3_(0 min) at ~270 °C (Peak 2, related to hydrogen interaction with $O_{lat}^{-}$) decreases approximately 3.5 times after mechanical treatment, as can be seen from the corresponding decrease of parameter A. As shown earlier (Figure 7a), the magnitude of the sensor effect is sensitive to changes in Gaussian peak height. Therefore, a decrease in the absolute value of the sensor signal is expected, which is confirmed by the experimental results (Figure 5a).

The position of the Gaussian peak $T_{m}$ changes by approximately 25 °C, which should lead to a corresponding shift in the temperature of the sensor response maximum (Figure 6b). The experiment showed that the change in $T_{max}$ in the dependence θ(T) is about 45 °C (Figure 5a). This observation can be explained by the contribution of the change in parameter σ, which controls the magnitude of the temperature shift (see Figure 6c). During mechanical treatment, many vacancies with a wide distribution of states are formed. This leads to the broadening of the distribution of states of oxygen anion radicals adsorbed at these vacancies, thereby increasing the value of parameter σ.

The proposed approach is not limited to In_2_O_3_-based sensing layers and can be extended to other systems in which the sensing properties are governed by the interaction of a reducing gas with surface active oxygen species. Its application to other gas-sensing systems is possible provided that the following conditions are satisfied:

1. The target gas is a reducing gas such as CO or NH_3_.
2. The overall reaction scheme is identical for all systems compared and can be expressed as $Gas\;+\;O_{m}^{n-}\to \;Products\;+\;ke^{-}$, where *n*, *m*, and *k* are determined by the reaction stoichiometry.
3. The sensor belongs to the conductometric type. The dominant conduction mechanism is charge transport through grain-boundary potential barriers, such that the proportionality $E_{b}\;\sim \;{\left(N_{O_{m}^{n-}}\right)}^{2}$ remains valid.
4. The surface and bulk contributions to the reduction profile can be distinguished and separated.
5. If the reduction profile contains multiple surface-related components, each component should be assigned to a specific elementary reaction. Only those TPR profile components associated with the elementary processes responsible for the sensor response should be considered when comparing sensing properties.

Under these conditions, the approach remains applicable even when comparing systems with different types of surface-active centers (i.e., different values of *n* and *m* in the $O_{m}^{n-}$). This is because differences in their reactivity and reduction temperature ranges are embedded in each individual TPR profile component. Thus, the decisive factors for the comparative analysis of gas-sensing systems are not the chemical identities of the surface centers themselves, but the characteristics of the corresponding TPR profile components, namely the parameters of the fitted Gaussian peaks.

## 4. Conclusions

The pretreatment of In_2_O_3_ samples has been demonstrated to significantly affect the structure of TPR profiles. The qualitative changes occurring during pretreatment are interpreted based on an existing kinetic description of sensing processes involving electrons and oxygen vacancies. Optimal conditions are determined for sample preparation for studying the sensing effect.

A procedure is proposed in which a relationship is established between TPR profiles and hydrogen sensor response within a thermally activated grain-boundary barrier conduction model. Within this approach, the influence of changes in Gaussian parameters decomposed from the TPR curve on the temperature dependence of sensor response is modeled and compared with the experiment.

The proposed approach reproduces both the change in absolute sensor response and the temperature shift of the maximum upon changes in surface state for different experimental conditions. The results demonstrate that TPR analysis can be used to predict changes in the sensing properties of polycrystalline oxides.

## Figures and Tables

**Table 1:** Parameters of Gaussian peaks decomposed from the TPR profiles of samples In_2_O_3_(0 min) and In_2_O_3_(30 min) pretreated in argon at 300 °C. The data represents parameters of surface reduction-related components only.

		Parameter					
Peak	Sample	A		$T_{m}$		σ	
		Value	Error	Value	Error	Value	Error
1	0 min	0.0097	5.86 × 10^−5^	190.47	0.55	38.64	0.33
	30 min	0.011	3.34 × 10^−4^	180.66	0.35	30.47	0.32
2	0 min	0.036	1.5 × 10^−4^	268.45	0.10	31.69	0.10
	30 min	0.011	1.49 × 10^−4^	242.50	1.38	47.79	0.66

## Data Availability

The original contributions presented in this study are included in the article. Further inquiries can be directed to the corresponding author.
